# Efficacy of oral viscous budesonide to reduce dilation treatment after esophageal atresia repair: a retrospective study

**DOI:** 10.3389/fgstr.2024.1404292

**Published:** 2024-05-28

**Authors:** Cosimo Ruggiero, Giusy Russo, Denis Cozzi, Silvia Ceccanti, Chiara Scanziani, Danila Volpe, Paola Papoff, Mattia Spatuzzo, Vasiliki Spyropoulou, Salvatore Oliva

**Affiliations:** ^1^ Pediatric Gastroenterology and Liver Unit, Maternal and Child Health Department, Sapienza – University of Rome, Rome, Italy; ^2^ Pediatric Surgery Unit, Maternal and Child Health Department, Sapienza – University of Rome, Rome, Italy; ^3^ Pediatric Intensive Care Unit, Maternal and Child Health Department, Sapienza – University of Rome, Rome, Italy; ^4^ Division of Pediatric Gastroenterology and Nutrition, University Children’s Hospital Zurich, Zurich, Switzerland

**Keywords:** esophageal atresia, topical steroids, stricture, dilation, symptom recurrence

## Abstract

**Introduction:**

Anastomotic stricture is a common complication following esophageal atresia (EA) repair, substantially affecting the patient’s quality of life (QoL). Multiple dilations are often required to maintain the appropriate diameter of the esophagus, leading to ongoing challenges. The aim of this study is to assess the efficacy of oral viscous budesonide (OVB) in prolonging the time between symptom recurrence and subsequent dilation.

**Methods:**

We carried out a retrospective single-center study for pediatric patients (0–18 years) who had undergone recurrent esophageal dilations (≥3) following EA repair and initiated treatment with OVB (1 mg/day <10 years, otherwise 2 mg/day). Efficacy of treatment was determined by assessing a dysphagia symptom score (DSS) ≤1 for at least 3 months. Recurrence time to dysphagia and dilation were analyzed according to Kaplan-Meier method.

**Results:**

Of 29 patients screened, 19 were enrolled: 19/19 were responsive to OVB and 13/19 (68%) didn’t required further dilations. The median time between dilations was significantly prolonged compared to the pre-treatment period [30 months vs 2 months; p<0.01] as well as the time to dysphagia relapse [18 months vs 1 month; p<0.01].

**Conclusion:**

Topical budesonide has proven to be an effective treatment for recurrent esophageal stricture in repaired EA. Further investigation is required to assess the long-term sustained response of symptoms to topical steroids.

## 1 Introduction

Esophageal atresia (EA) with or without tracheoesophageal fistula (TEF) is one of the most common congenital digestive anomalies (1, 2).

In recent decades, advancements in surgical techniques and perioperative care for newborns with EA have led to a remarkable survival rate, even in intricate cases. However, this progress has been accompanied by the emergence of long-term complications, presenting a significant challenge to clinicians. Among these complications, anastomotic stricture (AS) is the most common postoperative issue, affecting 18 to 60% of patients (2, 3).

AS manifests as a narrowing at the site of the esophageal anastomosis, proving clinically consequential in patients exhibiting esophageal dysfunction symptoms such as dysphagia, vomiting, regurgitation, feeding difficulties, and food impaction (2). While anastomotic dilatation using bougie or balloon dilators is the initial line of treatment for AS, it lacks a definitive solution, with over 50% of cases experiencing recurrence of stenosis (4, 5). Several non-surgical adjuvant treatments have been proposed for refractory and recurrent esophageal AS, including systemic steroid therapy (6, 7), intralesional steroid injection (8, 9) or mitomycin C (10, 11). However, these approaches have yielded somewhat unsatisfactory outcomes and notable side effects.

Topical administration of corticosteroids represents a cornerstone in treating eosinophilic esophagitis, a condition characterized by chronic tissue inflammation in the esophagus, potentially leading to esophageal remodeling and stenosis (12, 13). Self-made viscous solutions are prepared by combining budesonide nebules with sodium alginate or sucralose artificial sweetener. Moreover, oral viscous budesonide (OVB) has demonstrated an extended mucosal contact time in comparison to nebulized mode, yielding superior outcomes in terms of reducing inflammatory cell infiltration (14).

As for well-established anti-inflammatory effect of OVB, we hypothesize its potential in curbing AS post-esophageal atresia (EA) repair. This retrospective study focuses on EA patients who received adjunctive OVB following endoscopic dilation, aiming to assess its efficacy in promptly alleviating symptoms and reducing the need for re-dilations.

## 2 Methods

We conducted a retrospective cohort study at our tertiary referral center, the Pediatric Gastroenterology and Liver Unit – Sapienza University of Rome, spanning from January 2013 to May 2023. The study focused on pediatric patients (0–18 years) with repaired esophageal atresia (EA) who initiated topical steroid treatment due to recurrent anastomotic stricture, characterized by the inability to maintain a satisfactory luminal diameter after a minimum of 3 esophageal dilations (2). Patients were excluded in presence of other organic gastrointestinal diseases [eosinophilic esophagitis (EoE), inflammatory bowel disease (IBD), celiac disease, Helicobacter Pylori infection], a history of prior topical steroid or anti-fibroblast molecules injections, gastroesophageal reflux disease (as determined by pH-impedance: reflux index: >7%), and low compliance with treatments. To minimize the risk of associated peptic inflammation, all patients received a proton pump inhibitor (PPI; 1 mg/kg/day) following esophageal repair.

Data were collected from medical records, encompassing essential demographic characteristics such as age, sex, gestational age, birth weight, height, type of EA and associated malformations. Detailed information on the number and type of dilations, along with associated adverse events, both before and after the initiation of OVB treatment, was recorded. Dilation sessions were conducted using anterograde approach through pneumatic balloon or bougie dilators under general sedation.

The choice of method was determined by physician preference based on the age of the patient, position, and diameter of the anastomotic stricture observed during diagnostic esophagoscopy.

Pneumatic dilation balloon procedures were conducted by inserting a balloon catheter through the working channel and positioning it at the midpoint across of the anastomotic stricture. The balloon was then gradually inflated in 2 mm step-up increments, with each inflation lasting 60 seconds, until a reduction in tactile resistance was achieved. This process was repeated for a maximum of 3 times during the same session. Bougie dilations were performed using Savary-Gilliard bougies with a stepwise protocol, reaching a final diameter at discretion of endoscopist as deemed appropriate. Dilators were passed over an endoscopically placed guidewire, with 2 to 3 dilators being used each session, depending on the tightness of the stricture (2).

Following a series of dilation sessions (≥3), patients started adjuvant treatment with topical steroids at the last dilation for at least 3 months. If deemed effective, a one-year treatment plan was recommended. Patients were administered a self-prepared solution, consisting of a blend of 0.5 mg of inhaled budesonide and 5 mL of sodium alginate twice daily (1 mg/day) for those under 10 years rather 1 mg twice daily (2 mg/day) for those over 10 years (12). General clinical assessments were conducted every 3 months during and after OVB treatment. Dysphagia symptoms were graded using a previous published (15) 4-point scale [Dysphagia Symptom Score (DSS); 0= no dysphagia; 1= unable to swallow certain solids; 2= able to swallow only soft foods or experiencing food impaction; 3= only liquids are tolerated; 4= unable to swallow liquids]. The clinical efficacy of the topical steroid was assessed based on DSS of ≤1 after 3 months of treatment or in the absence of feeding difficulties for infants under 6 months of age. Subsequently, a new dilation was performed in presence of recurrent symptoms (DSS>1) during and after treatment. The interval from the last dilation, as well as dysphagia symptoms, was documented to assess any variation before and after OVB treatment. Influence and risk factors, including sex, age, duration of treatment etc. were analyzed to identify and eliminate potential confounding variable affecting the recurrence time of dilation. Safety assessments were conducted to identify any symptoms related to the operative endoscopy, and cortisol blood levels were monitored to estimate adrenal suppression with topical steroids during follow-up visits.

This study aligns with the ethical principles articulated in the Declaration of Helsinki and has received approval from the Sapienza University Ethics Committee.

Data were expressed as median values and interquartile ranges (IQR) for continuous variables, while frequencies were used for categorical data as appropriate. The comparison of continuous variables employed either the student t test or the Mann-Whitney for nonparametric distributions. Nominal and independent variables based on sample size frequencies were analyzed using Chi square or Fisher exact tests; in cases where appropriate, the McNemar test was applied. For repeated observations with non-parametric distribution, Friedman analysis or Wilcoxon signed-rank test were conducted to evaluate differences between values. The time to recurrence of symptoms and dilations was analyzed using Kaplan-Meier methods, with differences between the curves evaluated using the log-rank test. Subsequently, regression analysis, employing Cox regression to determine influence and logistic regression to determine risk factors (including age, sex, prematurity, type of atresia, number of dilations before OVB, and duration of treatments) was conducted. Statistically significance was established if the null hypothesis could be rejected with greater than 95% confidence (p<0.05). All statistical analyses were performed using SPSS Statistics v. 27 (IBM Corp., Armonk, N.Y., USA).

## 3 Results

**Figure 1:**
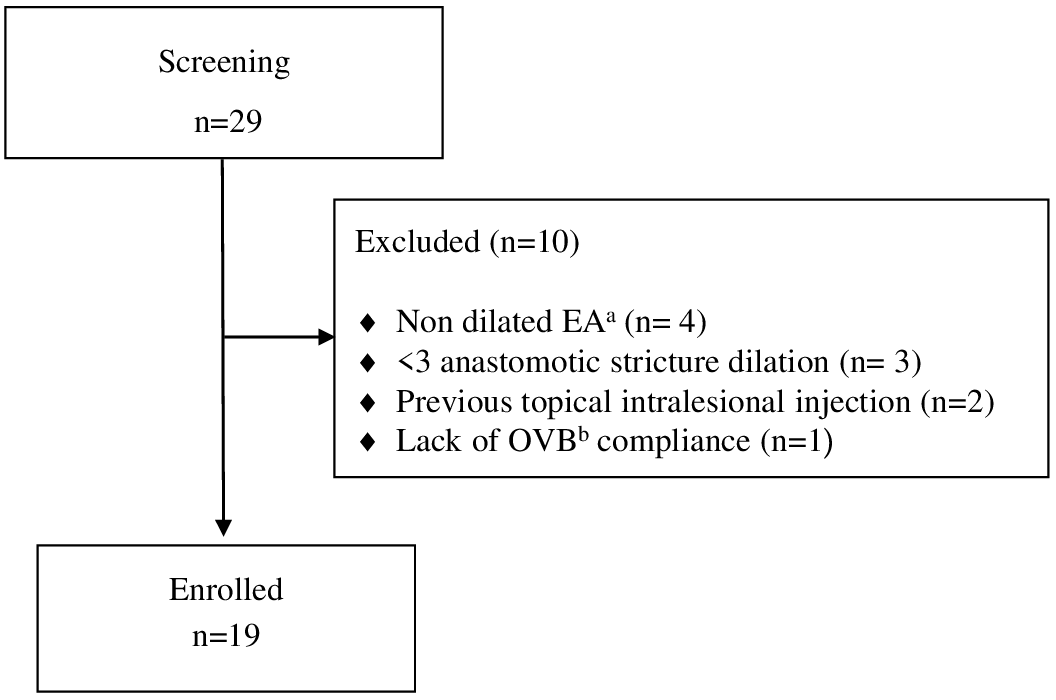
Flow diagram of study participants. ^a^ Esophageal atresia; ^b^ oral viscous budesonide.

Among 29 patients screened with EA, 19 patients underwent endoscopy-guided esophageal dilatation following EA repair due to recurrent stricture and initiated OVB treatment (**Figure 1**). The demographic characteristics of these patients are summarized in **Table 1**.

**Table 1:** Demographic characteristic of patients.

Characteristic	N=19
Age (m), median (IQR)	27 (19–52)
Sex (M, F)	12, 7
Gestational Age, n (%)	
• Preterm • Term • Post-term	6 (32%) 12 (63%) 1 (5%)
Birth weight (g), median (IQR) • < 1.5 Kg • 1.5 – 2 Kg • > 2.5 Kg	2640 (2192–3392) 2 (10%) 4 (21%) 13 (68%)
Type of Atresia, n (%) • A • B • C	2 (10%) - 17 (90%)
Postoperative complications • Fistula • Anastomotic leak	1 (5%) 2 (10%)
Early stricture anastomosis, n (%)	9 (47%)
Associated Malformation, n (%) • Cardiac defects • VACTERL association • Treacher Collins syndrome • Microduplication Xp11.22p11.23 • Hypoplasia of the corpus callosum • Duodenal atresia • Brachydactyly • Kidney cyst • Hypospadias	15 (79%) 5 3 1 1 1 1 1 1 1

a VACTERL, vertebrae, anus, heart, trachea, esophagus, kidney and limbs.

A total of 196 dilatations were performed prior to the commencement of OVB, with a median number of sessions per patient being 9 (IQR 5–12). Among these, 149 (76%) dilations were conducted using Savary-Gilliard dilators, and 47 (23%) were performed with balloons. The first endoscopy assessment post-surgery repair and dilation occurred at a median time of 6 months age (IQR, 4–8). During this assessment, 17 (90%) patients reported dysphagia or feeding difficulties, with associated symptoms including failure to thrive (5/19; 26%), regurgitation (4/19; 21%), vomiting (4/19; 21%), and chronic cough (2/19; 10%). The median time to recurrent dilation and symptom recurrence was 2 months (IQR, 1–3) and 1 month (IQR, 1–2), respectively. Dilation session proved clinically effective in 3/19 (15%) patients before the initiation of OVB. Food impaction was observed in 7 (36%) patients before OVB treatment, with 4 episodes presented in one case, 2 episodes in three cases and 1 episode for other three cases. Endoscopic removal was required for all patients experiencing food impaction.

Following a sequential dilations period of 17 months (IQR: 10–45), patients started topical steroid treatment for a median duration of 12 months (IQR: 8–12). The daily dose of OVB administered during this period was 1 mg (IQR1–1.5). Subsequently, patients stopped treatment and were monitored for 18 months (IQR 13–26) (treatment vs post-treatment time, p<0.01).

Topical steroid proved higher clinically effective as adjuvant treatment after 3 months compared to previous dilation session [n (%) =19 (100%), DSS= 0 (0–1) vs n (%) = 3 (15%), DSS=2 (1–2), respectively; p<0.001].

The median time of recurrence dysphagia was 18 months (IQR: 6–30) from the initiation of OVB treatment, in contrast to pre-treatment periods [1 month (IQR) 1–2); p < 0.01]. During and after OVB discontinuation, 9 (47%) patients experienced a recurrence of swallowing symptoms [during (n=4, 21%) and after (n=5, 26%) respectively], with regurgitation being the main associated symptom (21%), followed by respiratory symptoms (15%) and vomiting (5%). No patient underwent anti-reflux surgery for the symptoms presented during the study period. Two patients (10%) presented one episode of food impaction, while one patient (5%) had 4 episodes and required endoscopic removal in one event.

The median time of subsequent dilation was 30 months (IQR: 12–39) from the beginning of OVB treatment, as opposed to the pre-treatment period [2 months (IQR:1–3); p < 0.01]. The median number of dilations required was significantly lower during OVB treatment compared to the pre-treatment period [(median, 0 vs 9; p< 0.05). Two patients (10%) underwent dilation at 6 months of treatment, while 4 (21%) patients required dilation after OVB discontinuation (n=2, 18 months; n=1, 24 months, and n=1, 48 months) and all of them were patients with early anastomotic stricture (n=6/9, 66%). Subsequently, a new cycle of OVB was initiated in 4 patients, demonstrating clinical and sustained dilation efficacy.

**Figure 2:**
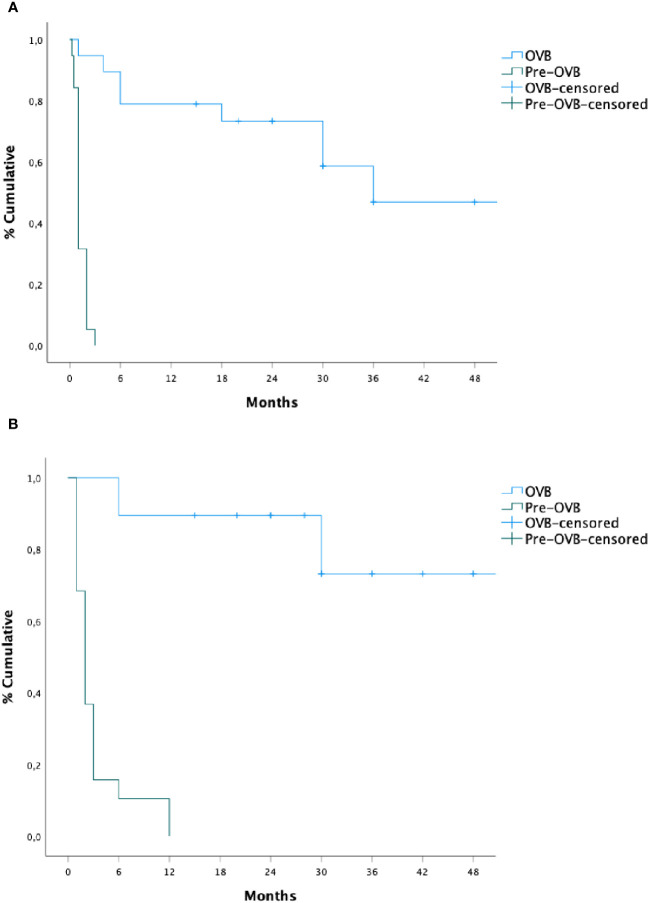
Recurrence time of subsequent dilation **(A)** and dysphagia **(B)** before and after oral viscous budesonide.

The cumulative probability of persistent clinical benefit was higher compared to pre-steroid phase, as well as for lower dilations needed (p= 0.001, **Figure 2**). No influencing factors on the time of dilation or risk factors for stricture relapse were observed after OVB treatment (**Table 2**).

**Table 2:** Correlations of influence and risk factors to recurrence dilations.

	Cox Regression			Logistic Regression	
*Variables*	*p-value*	*HR (CI, 95%)*	*Variables*	*OR (CI, 95%)*	*p-value*
Age	0.066	1.01 (0.99–1.02)	Age	1.01 (0.99–1.03)	0.27
Sex	0.509	0.47 (0.05–4.29)	Sex	0.81 (0.15–6.14)	0.83
Duration of treatment	0.52	1.05 (0.91–1.23)	Premature	0.88 (0.11–7.01)	0.91
			Type of Atresia	–	1
			Early Anastomosis	0.31 (0.04–2.38)	0.26
			Dilations	0.18 (0.92 – 1.51)	0.18

a OR, odds ratio; HR, hazard ratio; CI, confidence interval.

No adverse events (AE) were recorded during dilation sessions associated with topical steroid treatment [vs pre-OVB, n=15/196 (7%)]. The cortisol blood levels remained within the normal range throughout the study period for all patients receiving treatments. Only two (10%) patients experienced drug-related side effects: Candida infection, which was effectively managed. Treatments were resumed after a 15-day course of antifungal treatment without recurrency of symptoms.

## 4 Discussion

This study represents the first evaluation of the efficacy of OVB therapy in reducing both the frequency and the time to dilation, as well as symptom recurrence, in children with repaired EA and recurrent AS.

Following 3 months of treatment, all 19 patients responded positively to OVB, resulting in a dilation-free period. Notably, 13 of these patients (68%) did not require further procedures. Despite a longer interval since the last dilation, the number of dilations significantly decreased after treatment, particularly among patients with early anastomotic stricture, emphasizing the necessity for strict follow-up in this patient cohort. The presence of early dysphagia during dilation sessions alone reduced the observation time in the post-treatment phase, with a longer time to symptoms recurrence during and after OVB. AS presentation strongly correlated with dysphagia, with 66% of patients experiencing this symptom before undergoing dilation treatment at various time points. Only 2 patients (10%) underwent dilation during OVB, including those with previous long-gap atresia. Moreover, the number of patients requiring dilation doubled after a substantial period from treatment discontinuation, suggesting a sustained effect of topical steroids on stricture remodeling and supporting the recommendation for maintenance dosing. While various steroid applications in children with EA have been studied, our results demonstrate a lower number of dilations and a higher proportion of patients free from dilation compared to previous studies. In a retrospective study (16), 50% of patients (3 out of 6) required dilation within the first year after triamcinolone acetonide injection, showing a higher incidence compared our study [2/19 (10%)]. Additionally, only half of the patients (50%) were free from dilation during the same observed period, contrasting our better results [17/19, (89%)]. However, it is important to note that the potential benefits of this treatment have not been conclusively defined yet: indeed, a randomized controlled study in a pediatric population is currently underway (8). Injection of topical steroids have been used in adults, yielding diverse outcomes, with a superior success rate noted for caustic strictures compared to anastomotic ones (17–20). Consequently, the topical injection of steroids is currently not recommended (2) even in cases of refractory stenosis where mitomycin C has shown better results (10), but typically after multiple applications (11). Ley et al. (21) reported a success rate of 68% (17/25), defined by the reduction in dilations over the same period but with lower responsiveness (41%) in a similar long-term follow-up within EA population. It is noteworthy that our patients underwent treatment for a more extended period, using only OVB, and experienced fewer related adverse effects. Strict surveillance is advisable due to the higher risk of dysplastic lesions associated with mitomycin C injection (22), a risk already elevated in EA patients for esophageal carcinoma (23, 24). All our patients received PPI treatment throughout the study period to mitigate persistent acid exposure on stenosis (25) as this intervention did not influence the number of dilations or the incidence of strictures over time in comparative studies (26, 27). Despite the variable treatment durations within our cohort, we did not assess potential influences on the time of dilation relapse. The associated risk of recurrence stenosis was not assessed considering baseline factors and only one study noted a higher risk of recurrence in patients with early evidence of stricture formation (<29 days) (28). Topical steroids were well tolerated, with only 2 patients discontinuing treatment due to oral Candida infection, which was promptly managed. No patients experienced adrenal insufficiency or abnormal cortisol blood levels, indicating the reproducibility and safety of the dosage and formulation used. The number of peri-procedural adverse events was higher during the pre-phase treatment with OVB. However, the positive effect of significantly fewer dilations post-OVB requires further clarification. The pathological mechanisms of AS formation remain undefined, and the wide-ranging effect of steroids on secondary inflammation is postulated to reduce fibrotic healing. Except for EoE treatment in repaired EA (29), the administration of OVB for the recurrence of anastomotic stricture has not been previously explored, adding novelty to our findings. Moreover, the effect of topical steroids in reducing the presentation of anastomotic stricture immediately after surgery requires further evaluation.

This study has several limitations. Firstly, it is a single center study with a limited sample size and lacks a control group for comprehensive comparison. Nonetheless, all patients demonstrated sustained responsiveness during treatment, contributing to an improved quality of life. Secondly, our treatments were administered to patients experiencing recurrent symptoms related to esophageal dysfunction in anastomotic stenosis after a long time from repair. Further exploration is warranted to assess its potential as a preventive treatment for primary esophageal anastomotic stricture in infants, and its effectiveness for other types of strictures. Additionally, patients received treatments at varying times, employing different dose regimens and durations. Nevertheless, doses of topical budesonide were tailored based on age and administered for a minimum period of three months (12).

In conclusion, topical budesonide emerges as an effective and safe treatment for recurrent esophageal stricture in children with repaired esophageal atresia. Further evaluation of sustained topical steroids on symptom response is imperative for comprehensive understanding and guidance.

## Data availability statement

The raw data supporting the conclusions of this article will be made available by the authors, without undue reservation.

## Ethics statement

The studies involving humans were approved by University Hospital Umberto I of Rome - Italy. The studies were conducted in accordance with the local legislation and institutional requirements. Written informed consent for participation was not required from the participants or the participants’ legal guardians/next of kin in accordance with the national legislation and institutional requirements. Written informed consent was obtained from the minor(s)’ legal guardian/next of kin for the publication of any potentially identifiable images or data included in this article.

## Author contributions

CR: Conceptualization, Data curation, Formal analysis, Investigation, Methodology, Writing – original draft. GR: Investigation, Methodology, Writing – original draft. DC: Investigation, Writing – original draft. SC: Investigation, Writing – original draft. CS: Data curation, Writing – original draft. DV: Data curation, Writing – original draft. PP: Investigation, Writing – original draft. MS: Investigation, Writing – original draft. VS: Data curation, Writing – original draft. SO: Conceptualization, Investigation, Methodology, Writing – original draft, Writing – review & editing.
